# Case Report: Rare Presentation of Mixed Germ Cell Tumor in an Infant

**DOI:** 10.3389/fped.2021.729917

**Published:** 2021-09-07

**Authors:** Sriharsha Talluri, Michael A. Goedde, Susan Coventry, Eran Rosenberg, Katie L. Canalichio, Dennis Peppas, Jeffrey T. White

**Affiliations:** ^1^Department of Urology, University of Louisville, Louisville, KY, United States; ^2^Department of Pediatric Anatomic Pathology, Norton Healthcare, Louisville, KY, United States; ^3^Department of Pediatric Urology, Norton Healthcare, Louisville, KY, United States

**Keywords:** pediatric urology, germ cell tumor, testicular tumor, pediatric cancer, surgery

## Abstract

The estimated incidence of pediatric testis tumor is 0.5–2.0 per 100,000 children, accounting for 1–2% of all pediatric tumors. Mixed germ cell tumors (MGCT) in prepubertal males are exceedingly rare, with only one previous case report found in the literature. We report a case of a MGCT in an infant. For prepubertal males, GCTs typically present with a painless scrotal mass, though trauma, testis torsion and hydrocele are also common presentations. Similar to such tumors in postpubertal males, ultrasonography, computed tomography, and tumor markers are integral to determine the best treatment. The patient described in this report presented with a painless scrotal mass. Following orchiectomy, the patient was found to have MGCT that was limited to the testis. With prudent management, these patients tend to have favorable prognoses.

## Introduction

Prepubertal testicular tumors are rare. The total incidence of all testicular tumors is modeled with a bi-modal distribution, most prevalent within the first 2 years of life and young adulthood (1). Pediatric testis tumor incidence is reported as 0.5–2.0 per 100,000 children; testis tumors account for 1–2% of all pediatric tumors (2). The most common testicular tumors in adults are seminomas and mixed germ cell tumors (MGCT). The most common testis tumor in children is yolk sac tumor (YST). There is one previous report that discusses a case of MGCT in prepubertal children in depth (3), though one series did mention a few cases (4). Despite the differing incidence in tumor types between adults and children, general management of pediatric testicular tumors follows a similar algorithm (1). We present an extremely rare case of a MGCT in an infant with only one other case in the literature previously reported.

## Case Description

**Figure 1:**
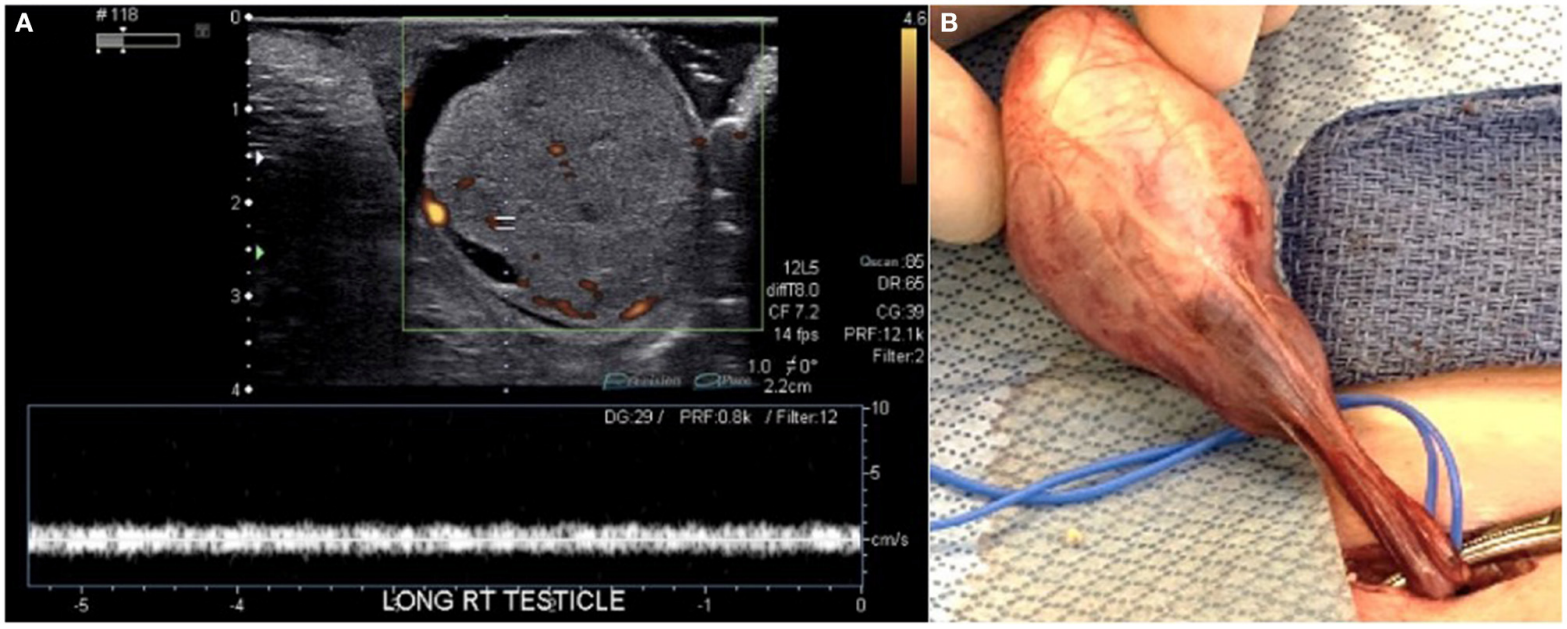
**(A)** Color Doppler ultrasonography showed an enlarged, heterogeneous, macronodular right testicle with cystic areas. Both arterial and venous flow were preserved, but hyperemia of the abnormal testis was present. **(B)** The testis and spermatic cord were accessed *via* an inguinal incision.

**Figure 2:**
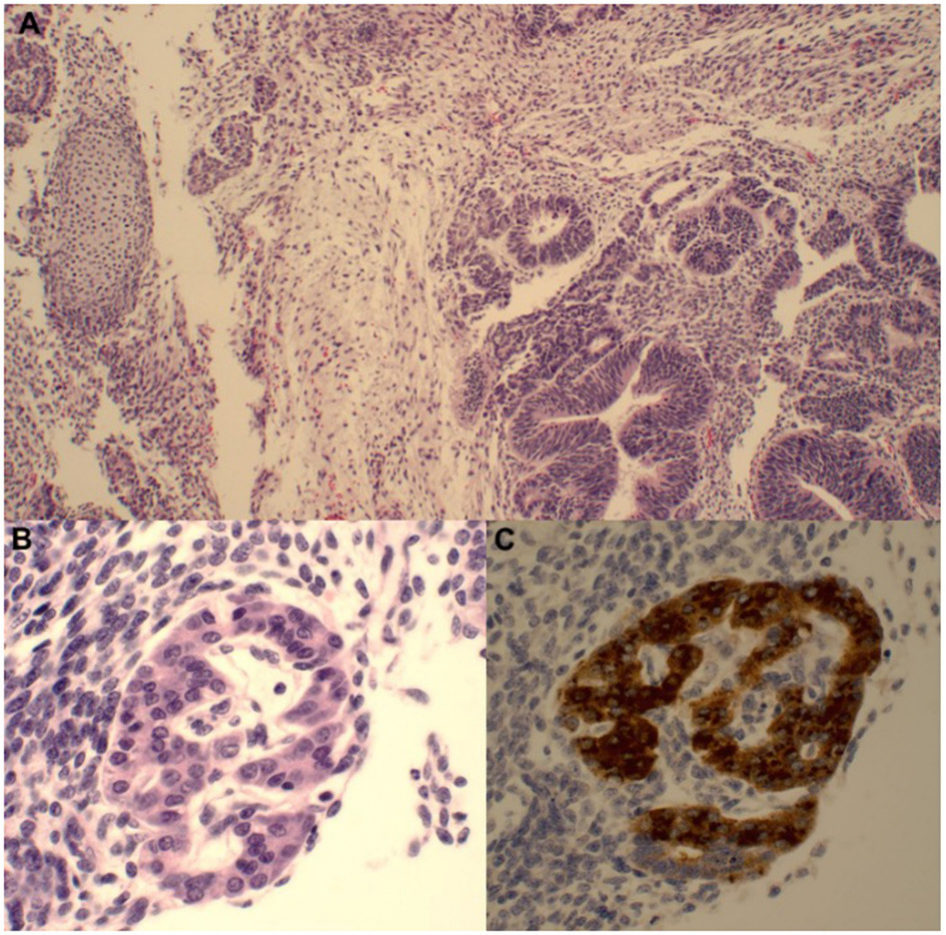
**(A)** The majority of the tumor comprises immature teratoma with a predominance of primitive neuroepithelium (bottom right) and a smaller primitive mesenchymal component (nodule of immature cartilage on the left). **(B)** There were rare small foci of yolk sac tumor with primitive glandular structures as well as a hepatoid focus demonstrating cords of immature cells with more abundant eosinophilic cytoplasm and occasional small nucleoli. **(C)** This focus is strongly AFP-positive.

A 7-week-old male presented to the emergency department with a 1-day history of painless scrotal swelling. On examination, a firm, irregular, non-tender right testis was palpated, but he was otherwise healthy. This child had no prenatal history and was born at full term. His scrotal exam at birth was unremarkable. There was no prior family history of cancer or testis masses. There were no known prenatal exposures to carcinogens. Complete blood count and basic metabolic panel were within normal limits. Color Doppler ultrasound (Figure 1A) revealed an enlarged, heterogeneous, macronodular right testicle with cystic areas. Arterial and venous flow were preserved with hyperemia of the abnormal testis. Tumor markers were obtained. Beta-human chorionic gonadotropin (HCG) was <2.4 IU/L (Ref. <2.4 IU/L) and alpha-fetoprotein (AFP) was 1,400 ng/ml (Ref. <2,433 ng/mL); lactate dehydrogenase (LDH) was 759 U/L (Ref. 180–430 U/L), making it only the tumor marker that was found to be abnormal. The patient underwent a right radical orchiectomy *via* the inguinal approach (Figure 1B). The spermatic cord was ligated at the level of the internal inguinal ring. Patient had a normal postoperative course without complication. Pathology revealed immature teratoma >80% and yolk sac tumor <20%. Tumor was limited to the testis with no lymphovascular invasion (Figure 2). CT chest, abdomen, and pelvis was performed; no extratesticular masses or abnormalities were discovered. There was no evidence of metastatic disease. This patient was classified as AJCC stage 1 and planned for close surveillance. He is pending enrollment in COG protocol AGCT1531 with oncology. He will undergo monthly tumor markers. CT of the abdomen/pelvis and chest X-ray will be performed at 3 and 12-months post-orchiectomy. Testicular ultrasounds will be performed at 6 months and annually thereafter.

## Discussion

Germ cell tumors (GCTs) account for nearly 98% of all testicular tumors in adults (5). Those tumors that are classified as GCTs can be further broken down into the following groups: germ cell neoplasia *in situ* (GCNIS), seminoma, and non-seminomatous germ cells tumors [spermatocytic, embryonal carcinoma, yolk sac tumor (YST), choriocarcinoma, and teratoma]. Some tumors can possess features of multiple types and are, therefore, designated as mixed GCTs (5).

Though testicular GCTs are most common following puberty, the distribution of GCTs is bimodal with a significant number occurring during the first year of life (6). The most common postpubertal GCT is YST followed by the mixed subtype (7), while the stratification for the prepubertal cohort is quite different. YSTs and teratomas are responsible for 49% and 13% of cases in the prepubertal patients, respectively. This specific study included adolescents into the category of pediatric tumors, with no prepubertal tumors being classified as MGCT or seminomas (1). A study by Ye et al., found that <4% of pediatric patients with testicular GCT had a MGCT (4). Recent publications have suggested that GCTs that arise in postpubertal patients should be stratified separately from prepubertal patients (8). In addition, GCNIS can be found adjacent to the majority of postpubertal GCTs; GCNIS is rarely found in association with GCT in prepubertal patients (9–11).

For prepubertal males, GCTs typically present with a painless scrotal mass, though trauma, testis torsion and hydrocele are also common presentations (12). A thorough physical exam is conducted to rule out other diagnoses such as inguinal hernia, epididymitis, or testicular torsion. Duplex Doppler ultrasonography should be utilized to characterize the lesion, allowing for a non-invasive assessment while also maintaining a sensitivity of nearly 100% in detecting GCTs (13). In addition to ultrasonography, tumor markers can prove beneficial. Elevated AFP levels can diagnose tumors with a yolk sac component; it is elevated in 90% of yolk sac tumors. This must be interpreted with caution: AFP can be naturally elevated during the first 6 months of life, allowing for benign masses to be mistaken for malignant tumors (1). A markedly elevated β-hCG level occurs in the setting of choriocarcinoma, although seminomas and embryonal carcinomas can have a modest increase as well (14). Rises in levels of lactate dehydrogenase (LDH) are not specific to one type of GCT but does indicate a larger tumor burden (15). Following orchiectomy, these tumor marker levels should be repeated monthly to ensure appropriate decreases and to monitor for disease recurrence.

The most common benign prepubertal masses are teratomas and epidermoid cysts. Prepubertal testis tumors are largely benign, thus surveillance with imaging or an excisional biopsy with frozen section analysis are viable options. There are multiple approaches to prepubertal masses, but surveillance or excisional biopsy may be preferred in all prepubertal tumors, except for those in children older than 6 months of age with an elevated alpha-fetoprotein. This preference is due to the high incidence of yolk sac tumors (1). Some urologists will approach a prepubertal testis tumor and normal tumor markers with a testis-sparing surgery and frozen section. If the frozen section reveals a benign histology, the testis can be spared. On the other hand, completion orchiectomy should follow if a malignant subtype is present in the frozen section (14). While teratoma is usually benign in adults, the majority of teratomas may harbor surrounding carcinoma *in situ* (CIS). This is in contrast to prepubertal children (16). Thus, prepubertal and postpubertal teratomas may require different management algorithms (17). The prognosis for prepubertal teratoma is favorable (18).

Many factors, such as tumor markers and risk factors for metastasis, affect the level of treatment that is needed for patients following resection. For this reason, the Children's Cancer Group/Pediatric Oncology Group (CCG/POG) has developed a staging system to help stratify tumors for management (Table 1) (19). For stage I YSTs and MGCTs in prepubertal boys, it is recommended that follow-up physical exams, tumor markers levels, and abdominal CT imaging be used to assess for recurrence. CCG/POG reported that this approach yielded a near 100% 5-year survival for Stage I patients (19). For prepubertal stages II–IV YSTs and MGCTs, surgical resection should be followed by a combination of bleomycin, etoposide, and cisplatin (BEP). If residual disease exists or tumor markers remain elevated, salvage chemotherapy and resection of remaining tumor should be considered (20). Though post-resection management for postpubertal YSTs and MGCTs is similar to that of prepubertal cases, there are a few differences. For stage I postpubertal tumors, surveillance is also the first line option (21). Like prepubertal tumors, stage II postpubertal tumors are best managed with BEP (22). However, one group that does have a different treatment for postpubertal patients is Stage IIa disease (with metastasis to lymph nodes that is <2 cm and involves ≤ 5 lymph nodes) with normal tumor markers. For these patients, the preferred treatment is retroperitoneal lymph node dissection (23).

**Table 1:** Children's Cancer Group/Pediatric Oncology Group (CCG/POG) staging system [modified from Schlatter et al. (19)].

**CCG/POG grading of testicular germ cell tumors**	
Stage I	• Limited to the testis • Removed in its entirety during high inguinal orchiectomy • Has an appropriate decrease in tumor markers following its removal
Stage II	• Invades to the scrotum and/or high spermatic cord • Has retroperitoneal lymphadenopathy∙ May be resected *via* transcrotalorchiectomy • Does not have an appropriate return of tumor markers to normal levels
Stage III	• Has advanced retroperitoneal lymph node involvement that is >2 cm wide • Has not spread to viscera or other areas of the body
Stage IV	• Presence of distant metastasis

We present a rare case of a MGCT in an infant. There is one other case report in the literature. The other case was histologically similar and comprised of teratoma and yolk sac tumor in a newborn (3). Despite its rarity, both were classified as Stage I tumors, in which the patients underwent active surveillance. With prudent management both patients should achieve favorable long-term outcomes. Further cases will need to be reported and current cases actively monitored to determine whether changes to therapeutic options are needed for Stage I testis tumors in prepubertal males.

## Data Availability Statement

The original contributions presented in the study are included in the article/Supplementary Material, further inquiries can be directed to the corresponding author/s.

## Author Contributions

All authors have made significant contributions to the manuscript including design, drafting and revising, and approved the final manuscript and its submission to Frontiers.

## Conflict of Interest

The authors declare that the research was conducted in the absence of any commercial or financial relationships that could be construed as a potential conflict of interest.

## Publisher's Note

All claims expressed in this article are solely those of the authors and do not necessarily represent those of their affiliated organizations, or those of the publisher, the editors and the reviewers. Any product that may be evaluated in this article, or claim that may be made by its manufacturer, is not guaranteed or endorsed by the publisher.
